# Presynaptic action of neurotensin on dopamine release through inhibition of D_2 _receptor function

**DOI:** 10.1186/1471-2202-10-96

**Published:** 2009-08-14

**Authors:** Charbel S Fawaz, Philippe Martel, Damiana Leo, Louis-Eric Trudeau

**Affiliations:** 1 Department of Pharmacology, Groupe de Recherche sur le Système Nerveux Central, Faculty of Medicine, Université de Montréal, Quebec, H3C 3J7, Canada

## Abstract

**Background:**

Neurotensin (NT) is known to act on dopamine (DA) neurons at the somatodendritic level to regulate cell firing and secondarily enhance DA release. In addition, anatomical and indirect physiological data suggest the presence of NT receptors at the terminal level. However, a clear demonstration of the mechanism of action of NT on dopaminergic axon terminals is lacking. We hypothesize that NT acts to increase DA release by inhibiting the function of terminal D2 autoreceptors. To test this hypothesis, we used fast-scan cyclic voltammetry (FCV) to monitor in real time the axonal release of DA in the nucleus accumbens (NAcc).

**Results:**

DA release was evoked by single electrical pulses and pulse trains (10 Hz, 30 pulses). Under these two stimulation conditions, we evaluated the characteristics of DA D_2 _autoreceptors and the presynaptic action of NT in the NAcc shell and shell/core border region. The selective agonist of D_2 _autoreceptors, quinpirole (1 μM), inhibited DA overflow evoked by both single and train pulses. In sharp contrast, the selective D_2 _receptor antagonist, sulpiride (5 μM), strongly enhanced DA release triggered by pulse trains, without any effect on DA release elicited by single pulses, thus confirming previous observations. We then determined the effect of NT (8–13) (100 nM) and found that although it failed to increase DA release evoked by single pulses, it strongly enhanced DA release evoked by pulse trains that lead to prolonged DA release and engage D_2 _autoreceptors. In addition, initial blockade of D_2 _autoreceptors by sulpiride considerably inhibited further facilitation of DA release generated by NT (8–13).

**Conclusion:**

Taken together, these data suggest that NT enhances DA release principally by inhibiting the function of terminal D_2 _autoreceptors and not by more direct mechanisms such as facilitation of terminal calcium influx.

## Background

Neurotensin (NT) is a peptide originally isolated from bovine hypothalamus [1]. It is found in the CNS and gastrointestinal tract. In the CNS, NT acts as a neurotransmitter or neuromodulator and one of its better known actions is to modulate dopaminergic transmission within the mesolimbic and nigrostriatal pathways [2]. In addition, a number of studies suggest that NT may be implicated in the pathophysiology of CNS disorders including schizophrenia, Parkinson's disease and drug abuse [2-5].

Considerable efforts have been made to characterize how NT acts to enhance dopamine (DA) release. When applied to the ventral tegmental area (VTA), NT increases the firing rate of DA neurons and DA release in terminal fields of the nucleus accumbens (NAcc) and the prefrontal cortex[6-8]. Moreover, acute microinjection of NT into the VTA enhances motor activity and facilitates DA-dependent behaviors[5,6,8]. At a mechanistic level, recent work has established that somatodendritic NT receptors enhance the firing rate of DA neurons through a Ca^2+^-dependent mechanism [9].

At the terminal level, NT also acts to enhance DA release. First, anatomical evidence for a presynaptic localization of NT receptors has been provided [10-12]. Second, NT facilitates K^+^-evoked and electrically-evoked DA release in dorsal striatal slice preparations [13,14] as well as in dorsal striatum *in vivo *[15]. These results support a presynaptic effect of NT on DA neuron axon terminals, but previous studies have not identified the mechanism involved or excluded an indirect mechanism of action. Recent work studying glutamate cotransmission in cultured DA neurons failed to provide support for a direct excitatory effect of NT on axon terminals [16]. In addition, a previous preliminary report failed to detect an enhancement of DA release evoked by single electrical pulses in NAcc slices [17]. Thus, the mechanism of action of NT on dopaminergic axon terminals remains unclear.

In the present study, we used fast-scan cyclic voltammetry (FCV) to better characterize the presynaptic action of NT in the NAcc. We find that although NT fails to increase DA release evoked by single pulses, it strongly enhances DA release evoked by pulse trains that lead to prolonged DA release and engage D_2 _autoreceptors. Our results suggest that NT acts to enhance DA release by inhibiting the function of terminal D_2 _autoreceptors.

## Results

### Time course of DA overflow evoked by single-pulses and train-pulses in the nucleus accumbens

**Figure 1:**
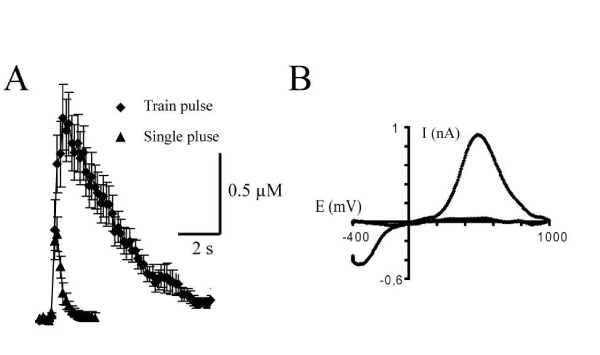
**Time course of evoked DA overflow in the nucleus accumbens**. A) Average evoked DA overflow following single-pulse (400 μA, 1 ms/pulse, n = 5) and train-pulse (400 μA, 0.1 ms/pulse, 30 pulses at 10 Hz, n = 5) stimulations. B) Sample cyclic voltammogram. The voltammogram was obtained at the peak of evoked DA overflow under control conditions. The positions of the oxidation and reduction peaks are compatible with the fact that the species detected was DA.

**Figure 2:**
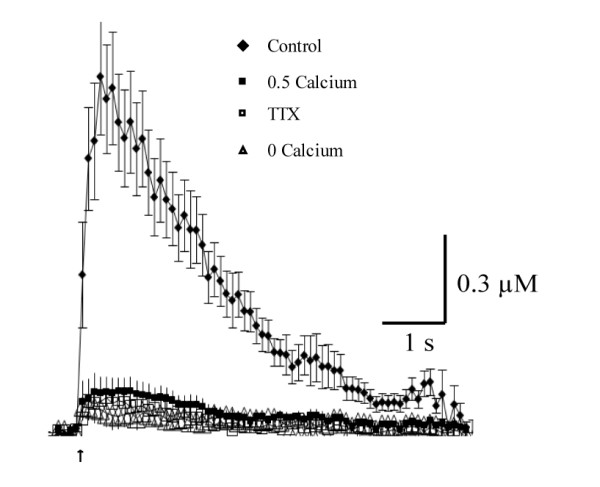
**Dependence of DA overflow on cell firing and extracellular calcium concentrations**. Graph comparing the time course of DA overflow following train-pulse stimulations under control conditions (2.4 mM calcium) (n = 5) or in the presence of 1 μM TTX (n = 3), 0.5 mM calcium (n = 6) or 0 mM calcium (n = 2).

Fast-scan cyclic voltammetry was used to monitor DA release at high time resolution in the shell and core/shell border region of Sprague-Dawley rat NAcc slices. We recorded DA overflow following single-pulse (400 μA, 1 ms) and in response to train-pulse stimulations (30 pulses delivered at 10 Hz), a frequency that can occur during bursting *in vivo *(Fig. 1A). The released substance was identified as DA since the oxidation currents recorded corresponded to the peak oxidation potential for DA (~+600 mV) (Fig. 1B). With train stimulation, DA concentrations peaked at 1.13 ± 0.18 μM (n = 5). DA concentrations rose to a maximum within ~400 ms (n = 5). The return to baseline was reached approximately 6 seconds after the peak signal and the signal width at half-height was ~2.2 s (n = 5). With single-pulse stimulation, DA levels reached a peak at 0.48 ± 0.09 μM (n = 5). DA concentrations rose to their maximum within ~200 ms (n = 5). The return to baseline was observed approximately one second after the peak and the signal width at half-height was ~0.4 s (n = 5). A significant difference was detected between the two stimulation conditions for the peak DA levels reached (Student's *t*-test, t = 5.07, p < 0.001). As expected, the release of DA evoked by electrical stimulation of the NAcc was blocked by TTX (1 μM) (15.3 ± 8.2% of control, n = 3) and was thus action potential-dependent, as well as by the removal of extracellular calcium (17.2 ± 11.0% of control, n = 6, with 0.5 mM Ca^2+^; and 10.5 ± 6.4% of control values, n = 2, with 0 mM Ca^2+^) (Fig. 2).

### NT facilitates DA overflow evoked by train-pulses but not by single-pulses

**Figure 3:**
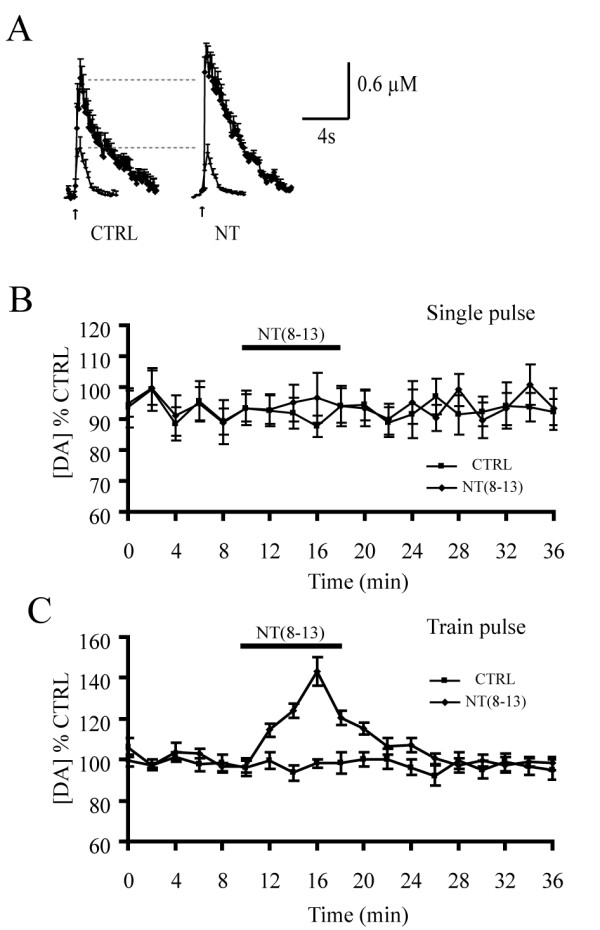
**Neurotensin facilitates train pulse- but not single pulse-evoked DA overflow in the NAcc**. A) Graphs representing the average amplitude and kinetics of single-pulse (lower traces) and train-pulse (upper traces) evoked DA overflow in the presence of NT (8–13) (100 nM) compared to control. B) Time course of peak DA overflow evoked by single electrical pulses. NT (8–13) (100 nM) was applied for a total of 8 min. It exerted no effect on single pulse-evoked DA overflow (n = 5). C) Time course of peak DA overflow evoked by train pulses. NT (8–13) strongly enhanced train-evoked DA overflow (n = 13). Data are expressed as a percentage of the DA levels recorded during the control period before NT.

We next evaluated the effect of NT on electrically-evoked DA overflow in the NAcc. Using single electrical pulses, we found that at a saturating concentration [9], NT (8–13) (100 nM) failed to alter DA overflow (94.4 ± 5.5% of control; Student's *t*-test, t = 0.23, p > 0.05, n = 5), suggesting that DA release is not directly facilitated by NT (Fig. 3A, B). In sharp contrast, using train-pulse stimulation, NT significantly enhanced peak DA overflow to 143.1 ± 6.8% of control (Student's *t*-test, t = 3.98, p = 0.001, n = 13) (Fig. 3A, C). The facilitatory effect started to decline before the end of the period of agonist application: DA concentrations recorded after 8 min of NT application were lower than after 6 min (one-way ANOVA performed on time, T = 0 to T = 36, followed by *post hoc *Tukey tests, F = 13.81, p < 0.001). This observation is compatible with the known ability of type 1 NT receptors (NTR1) to desensitize in response to sustained stimulation [18]. In accordance with this, the preferential NTR1 antagonist SR42948 (1 μM) completely prevented the facilitatory effect of NT (peak overflow 100.8 ± 5.7% of control; t = 0.376 p > 0.05, n = 5) (results not shown).

### NT enhances DA release through inhibition of D_2 _autoreceptor function

**Figure 4:**
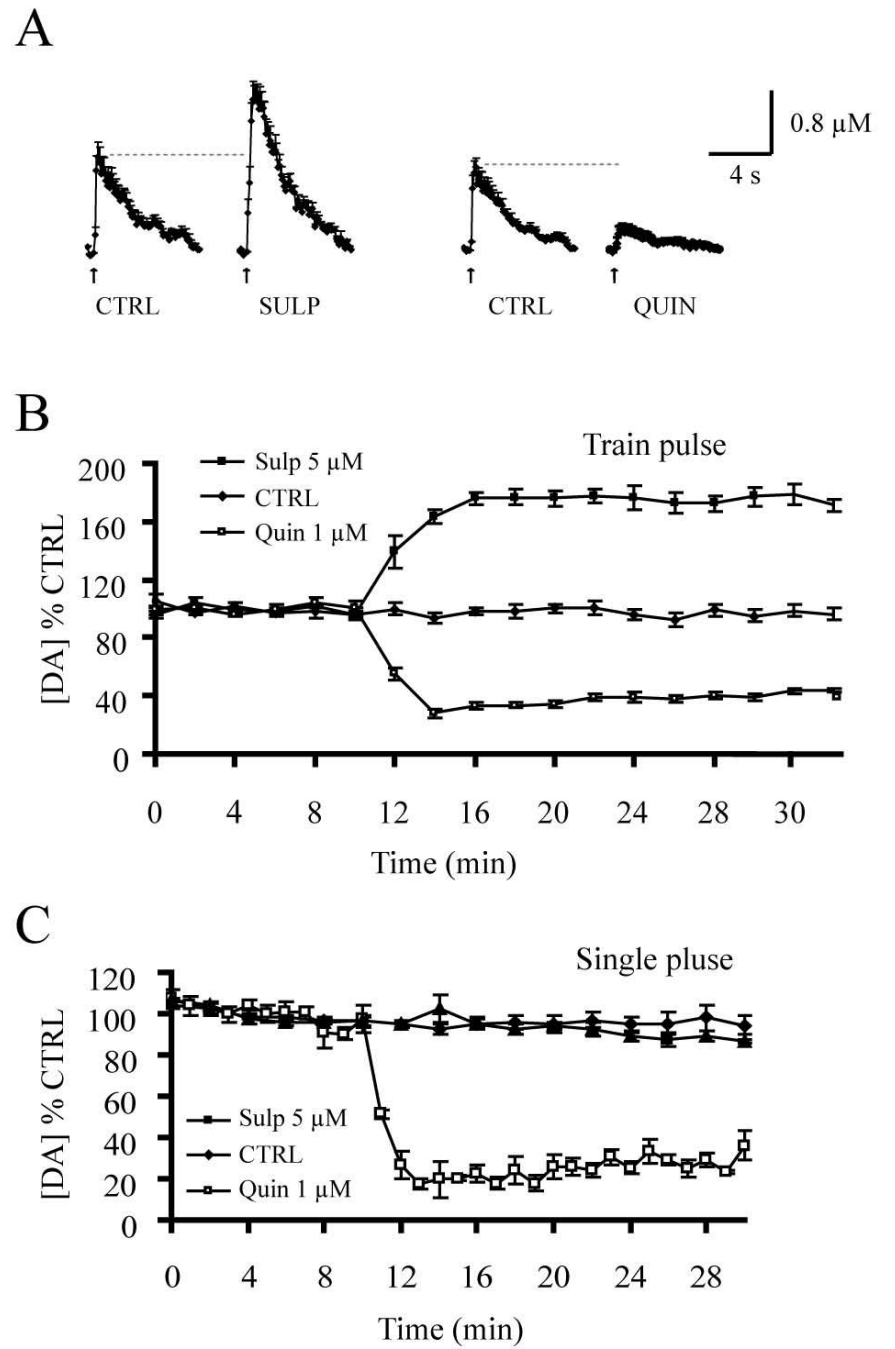
**Activation of D_2 _autoreceptors during train pulse- but not single pulse-evoked DA overflow**. A) The graphs represent the average amplitude and kinetics of train-evoked DA overflow in the presence of 5 μM sulpiride (left) or 1 μM quinpirole (right). B) Graph showing the effect of the D_2 _receptor agonist quinpirole (1 μM; n = 3), and the D_2 _receptor antagonist sulpiride (5 μM; n = 8) on DA overflow following pulse-train stimulation. Activation of D_2 _receptors by quinpirole inhibits peak DA overflow, whereas the blockade of these receptors by sulpiride considerably enhances it. C) Graph comparing the effect of quinpirole (1 μM; n = 5), and sulpiride (5 μM; n = 4) on DA overflow evoked by single-pulse stimulation. Under these conditions, D_2 _receptor activation also inhibits DA overflow, but D_2 _receptor blockade has no effect.

Previous work has suggested that NT may decrease terminal D_2 _receptor function [16,19-21]. One interpretation of the ability of NT to facilitate train-evoked but not single pulse-evoked DA overflow is therefore that NT acts principally by inhibiting the function of terminal D_2 _autoreceptors. This would imply that contrarily to train stimulation, D_2 _autoreceptors are usually not activated in response to single pulse stimulation, as was previously demonstrated [22-25]. To evaluate this hypothesis under our conditions, we next quantified evoked DA overflow using selective agonists and antagonists of the D_2 _autoreceptor. The D_2 _receptor agonist quinpirole (1 μM) decreased pulse train-evoked DA overflow to 27.9 ± 2.5% of control (Student's *t*-test, t = 12.33, p < 0.001, n = 3) (Fig. 4A, B) and single pulse-evoked DA overflow to 19.8 ± 8.7% of control (Student's *t*-test, t = 10.24, p < 0.001, n = 4) (Fig. 4C). In contrast, the D_2 _receptor antagonist sulpiride (5 μM) enhanced DA release triggered by pulse trains (175.3 ± 4.1% of control; Student's *t*-test, t = 14.4, p < 0.001, n = 8) (Fig. 4A, B), but had no effect on DA overflow evoked in response to single pulses (87.3 ± 1.7% of control values, Student's *t*-test, t = 1.40, p > 0.05, n = 4) (Fig. 4C).

**Figure 5:**
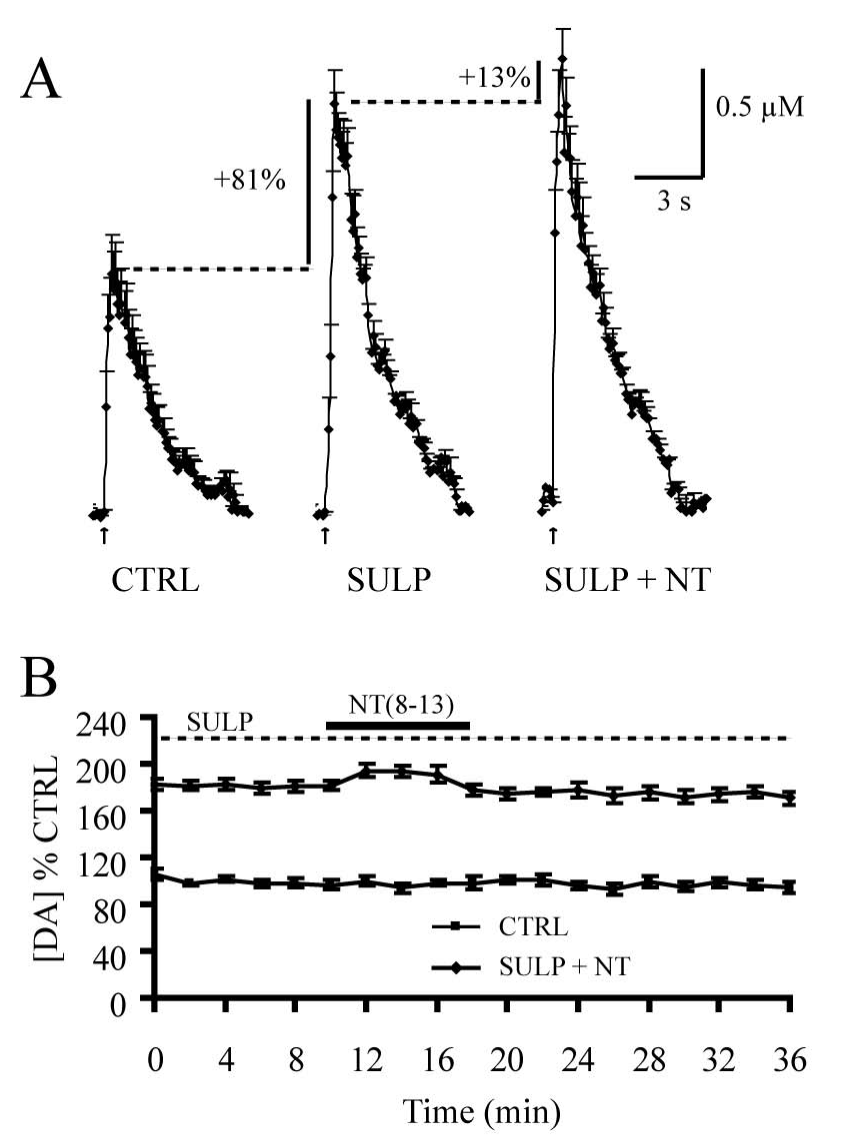
**NT enhances train-pulse evoked DA overflow through inhibition of D_2 _autoreceptor function**. A) Sulpiride (5 μM) alone was first pre-applied. With sulpiride present in the bath, NT (8–13) (100 nM) was introduced for a total of 8 min. The graphs represent the average amplitude and kinetics of train pulse-evoked DA overflow recorded during the control period (left), in the presence of sulpiride alone (middle) and in the presence of both NT (8–13) + sulpiride (right) (n = 10). Sulpiride alone enhanced peak DA overflow by 81% compared to control. In the presence of sulpiride, NT (8–13) only caused a small additional increase in DA levels (by 13% compared to the sulpiride alone condition) (n = 10). B) Summary graph showing the time course of the effect of NT (8–13) on peak DA overflow evoked by train-pulse stimulation following D_2 _receptor blockade by sulpiride (n = 10).

We next tested whether NT still increased DA overflow following D_2 _receptor blockade. We first applied sulpiride (5 μM) alone and found that it increased DA levels to 181.5 ± 3.1% of control values (n = 10) (Fig. 5A). With sulpiride present in the bath, NT (8–13) (100 nM) produced only a minor additional increase in DA levels (to 194.1 ± 4.6% of the control level, n = 10), thus reflecting an almost complete block of the facilitating effect of NT (Fig. 5A, B). A one-way ANOVA with *post hoc *Tukey tests revealed a significant difference between control and these two conditions (sulpiride and sulpiride + NT) (F = 3.57, p < 0.05). A specific comparison of the enhancement of DA overflow by NT in the presence of sulpiride (12.7 ± 4.3% above sulpiride alone) or in its absence (43.1 ± 6.8% above control) shows that the effect of NT was strongly decreased under autoreceptor blockade (Student's *t*-test, t = 3.51, p = 0.002). These results suggest that the majority of the facilitatory effect of NT on DA release is mediated through inhibition of D_2 _autoreceptor function.

### Releasable pools of DA are not depleted in the presence of sulpiride

**Figure 6:**
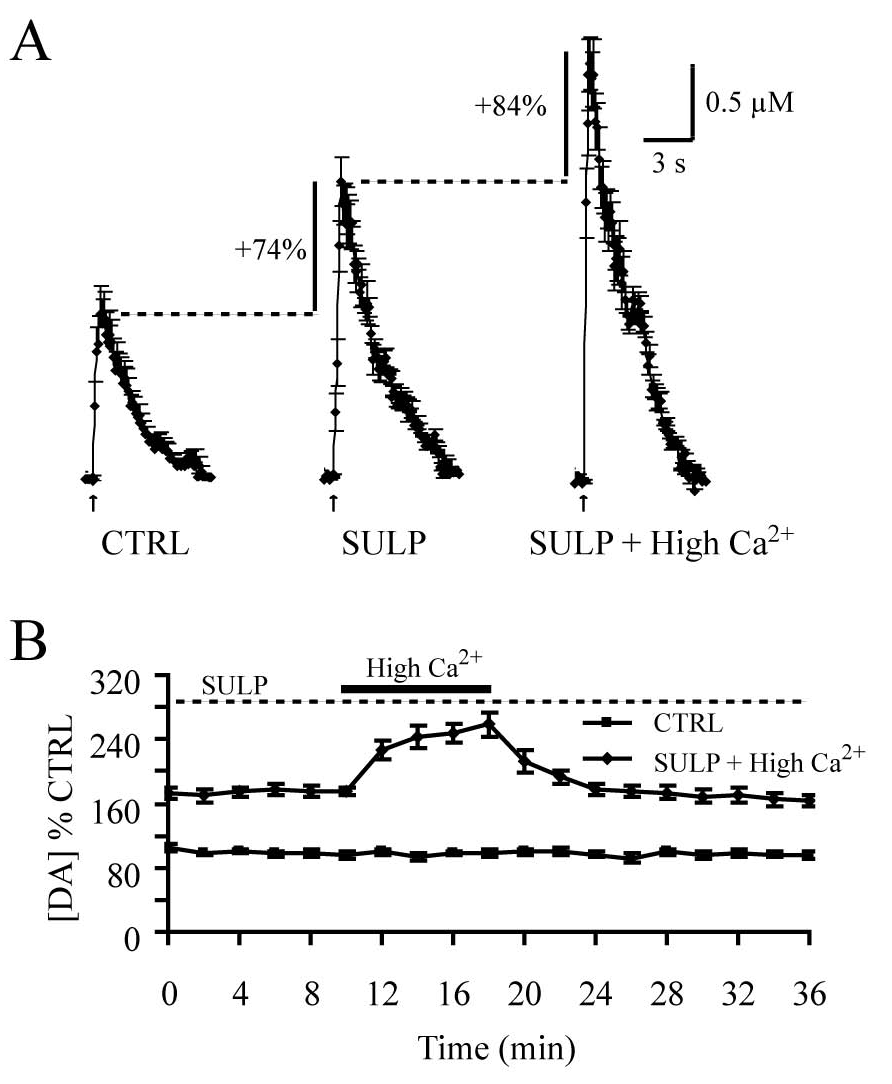
**Releasable pools of DA are not depleted in the presence of sulpiride**. A) The graphs represent the average amplitude and kinetics of train pulse-evoked DA levels measured in the presence of sulpiride (5 μM) alone (+74% above control, n = 7) or sulpiride in the presence of an elevated concentration of extracellular Ca^2+ ^(3.4 mM) (+ 84% above sulpiride alone, n = 7). B) Summary graph showing the time course of the effect of an elevation of extracellular Ca^2+ ^to 3.4 mM (for a total of 8 min) with pre-applied sulpiride (5 μM) in the bath (n = 7).

Pulse trains generate considerable DA release in the NAcc brain slice in the presence of sulpiride (maximal increase of 2.2 μM in the presence of both sulpiride and NT). The blockade by sulpiride of the ability of NT to facilitate DA release could thus in principle be an artefact resulting from a ceiling effect due to depletion of the releasable pools of DA or to the inability to further increase release probability. To test this possibility, we pre-applied sulpiride (5 μM) and then introduced a modified aCSF containing an elevated concentration of calcium (3.4 mM Ca^2+^). Sulpiride first increased DA overflow to 173.6 ± 6.9% of control levels (n = 7) (Fig. 6A). Elevation of extracellular Ca^2+ ^to 3.4 mM in the presence of sulpiride produced an additional increased to 258.1 ± 15.1% of control (n = 7) (Fig. 6A, B). A one-way ANOVA followed by a *post hoc *Tukey test confirmed significant differences between the control, sulpiride and 3.4 mM Ca^2+ ^conditions (F = 5.57, p < 0.001). The maximal DA release detected with sulpiride and elevated Ca^2+ ^exceeded 3.0 μM. Thus, the blockade by sulpiride of the ability of NT to facilitate DA release is not likely to simply result from a depletion of releasable pools of DA or to an inability to further increase release probability.

## Discussion

Although multiple aspects of the action of NT at the cell body level in the VTA have been investigated [26-28], the mechanism of action of NT on dopaminergic axon terminals is still unclear. The present results provide new insight into the mechanism mediating the facilitation of DA release by NT at the level of axon terminals in the NAcc. We show that this mechanism implicates a decrease by NT of the effectiveness of terminal D_2 _autoreceptors that normally inhibit DA release. We also demonstrate that the effects of NT on DA release differ greatly depending on the electrical stimulation parameters used to elicit this release. On the one hand, NT fails to alter DA overflow triggered by single pulses in the NAcc. On the other hand, NT strongly enhances DA release evoked by pulse trains that generate prolonged DA release and strongly engage D_2 _autoreceptors.

### An indirect mechanism can be discounted

NT is known to facilitate K^+ ^and electrically-evoked DA release in dorsal striatal slice preparations [13,14] as well as *in vivo *[15]. Although these observations imply a presynaptic effect of NT on dopaminergic axon terminals, previous results have not excluded an indirect mechanism of action. For example, NT could act through receptors located in the striatum on other elements than dopaminergic axon terminals. These receptors could for instance be located on corticostriatal glutamatergic axon terminals. If this were the case, NT could facilitate the spontaneous release of glutamate, which would then enhance DA release by depolarizing dopaminergic axon terminals. Although we cannot formally exclude this possibility or other possible indirect mechanisms, a major role for glutamate in mediating the ability of NT to enhance train-evoked DA overflow is not easily reconcilable with our observation that this facilitatory effect of NT is blocked in the presence of the D_2 _antagonist sulpiride. However, we cannot exclude a possible partial implication of postsynaptic D2 receptors located on NAcc medium spiny neurons, through some retrograde signaling mechanism.

### Action of NT on axon terminals

Previous experiments have explored the characteristics of DA D_2 _autoreceptors under different stimulation conditions varying the pulse/train duration or interpulse intervals, but didn't directly address the issue of the control of DA release by NT at the terminal level [22-25]. Nonetheless, a previous preliminary report showed that NT fails to enhance DA release evoked by single electrical pulses in NAcc slices [17], and suggested that DA release is not directly facilitated by NT. This previous report is consistent with our observation of an apparent lack of effect of NT on DA overflow evoked by single pulses. Although this finding could be taken as arguing against a role of terminal NT receptors in regulating DA release, our findings using train pulse-evoked release shed light on this paradox by showing that NT acts instead to enhance DA release by inhibiting the function of terminal D_2 _autoreceptors. Our results support a recent report in which the authors monitored glutamate co-release in cultured DA neurons and found that NT does not directly increase glutamate release, but rather attenuates the function of presynaptic D_2 _receptors that otherwise inhibit glutamate release [16].

Two of our present findings argue in favor of the hypothesis that NT mainly acts to enhance DA release by inhibiting the function of terminal D_2 _autoreceptors. First, we show that only pulse train-evoked DA overflow is facilitated by NT. This observation is compatible with previous reports showing that a minimum of 150–300 milliseconds is required for D2 autoreceptor activation to inhibit DA release, conditions that are satisfied during train stimulation, but are not optimal to inhibit release induced by single short pulses [22-25]. In our experiments, the lack of facilitation by sulpiride of single-pulse evoked DA overflow is in favor of this interpretation. Second, we demonstrate that the facilitating effect of NT on pulse train-evoked DA overflow is almost completely prevented by pre-blockade of D_2 _receptors. An alternate interpretation of this later finding is that in the presence of sulpiride, the releasable pools of DA become depleted during stimulus trains or the release probability cannot be further increased, thus masking any subsequent facilitating effect of NT. Considering our observation that elevating extracellular Ca^2+ ^strongly enhances DA overflow after pre-application of sulpiride, this hypothesis of a ceiling effect is unlikely. Nonetheless, we cannot completely exclude that releasable pools of DA are modified in elevated extracellular Ca^2+^. Alternate explanations of the ability of D_2 _receptor blockade to prevent the facilitating effect of NT on DA release thus cannot completely be discounted.

Together, our results thus suggest that in the NAcc, NT acts mainly by inhibiting the function of terminal D_2 _autoreceptors, leaving room for only a minor contribution of an additional mechanism. Although this second mechanism is presently unidentified, the capacity of NT receptors to mobilize intracellular Ca^2+ ^in DA neurons [9] leaves opens the possible implication of a Ca^2+^-dependent priming of synaptic vesicles. Although we have not directly examined the dorsal striatum in the present experiments, our results are compatible with previous biochemical and *in vivo *microdialysis work also suggesting that NT acts to inhibit the function of terminal D_2 _autoreceptors in the dorsal striatum [19-21,29].

In the present study, we found that the preferential NTR1 antagonist SR48692 prevented the facilitatory effect of NT on train-evoked DA overflow. Although we cannot exclude a partial contribution of the type 2 NT receptor (NTR2), our finding is compatible with previous data showing that DA neurons of the VTA and substantia nigra express abundant levels of NTR1 [30,31], but only modest amounts of NTR2 [32]. Second, the excitatory effects of NT on DA neurons are maintained in NTR2 knockout mice but strongly decreased in NTR1 knockout mice [33]. Compatible with this, SR48692 has been shown to block the ability of NT to increase the firing rate of DA neurons in culture [9]. Finally, SR48692 blocks the ability of NT to reduce the effect of the D_2 _agonist pergolide on extracellular DA levels in the striatum in microdialysis experiments [29].

## Conclusion

In summary, our results provide a better understanding of the mechanism of action of NT on dopaminergic axon terminals. We suggest that NT through its type 1 receptor enhances DA release mainly by inhibiting the function of D_2_-type autoreceptors, thus disinhibiting DA release. Future experiments should be oriented toward identifying the specific mechanism involved, such as heterologous desensitization of the D2 autoreceptor by NTR1 or direct receptor-receptor interactions.

## Methods

### Animals and slice preparation

Experiments were performed in accordance with the Université de Montréal animal ethics committee guidelines. Sprague Dawley rats, aged 4 to 6 weeks, were lightly anaesthetized with halothane and decapitated. Their brains were rapidly removed and transferred into ice-cold artificial cerebrospinal fluid (aCSF) containing: 125.2 mM NaCl, 2.5 mM KCl, 26 mM NaHCO_3_, 0.3 mM anhydrous KH_2_PO_4_, 2.4 mM anhydrous CaCl_2_, 1.3 mM anhydrous MgSO_4 _and 10 mM D-glucose. NAcc brain slices were then cut on a VT 1000s vibratome (Leica Microsystems, Nussloch, Germany) at a thickness of 400 μm. The brain slices were separated into right and left sections at the mid-sagittal line after transfer to a holding chamber. Slices were allowed to recover for 1 hour at room temperature in the holding chamber in oxygenated (95% O_2_, 5% CO_2_) aCSF and then transferred to a custom-built recording chamber and perfused (1 ml/min) with aCSF at 35°C. For experiments performed in the absence of extracellular calcium, the composition of the zero calcium saline was NaCl 140 mM, KCl 5 mM, MgCl_2 _4 mM, EGTA 1 mM, Hepes 10 mM, Sucrose 4 mM, Glucose 10 mM.

### Electrochemical recordings

Freshly-cut, disk carbon fibre electrodes of 5 μm diameter were fabricated according to procedures described by Kawagoe et al. [34] and by Kuhr and Wightman [35]. Electrodes were backfilled with a 4.0 M potassium acetate solution. The background current ranged from 50 to 180 nA. Fast-scan cyclic voltammetry was used to monitor DA release [36]. A triangular voltage waveform (-400 to +1000 mV at a rate of 300 V/s) was applied to the electrode every 100 ms and was computer-controlled using Clampex 9 software and a Digidata 1200B analog to digital converter (Axon Instruments, Union City, CA). This voltage ramp was applied to the electrode via an Axopatch 200B amplifier (Axon Instruments, Union City, CA) in voltage-clamp mode. The background-subtracted voltammograms were used to identify the released substance. Prior work has also established that the primary catecholamine released in the rat NAcc slice is DA [37]. Calibration was performed in vitro for each working electrode before and after the experiment in a 1 μM DA solution, quantifying the peak of the oxidation current after adding DA.

### Electrical stimulation

Single-pulse or train-pulse electrical stimulations were generated by an S-900 stimulator (Dagan, Minneapolis, MN) and computer-triggered. Stimulations were applied to the NAcc brain slice through a twisted bipolar tungsten stimulation electrode (Plastics One, Roanoke, VA). The 2 stimulating electrode tips were separated by 100 to 150 μm and were gently placed on the surface of the NAcc brain slice using a micromanipulator (Newport, Fountain Valley, CA). Single-pulse (1 ms) and train-pulse (10 Hz, 30 pulses, 0.1 ms/pulse) stimuli were then generated to evoke DA release. Pulse amplitude was 400 μA for both single and train pulses.

### Experimental design

The recording chamber was connected to a temperature-controlled perfusion and aspiration system. The carbon fibre electrode was then inserted ~75 μm into the slice by a piezoelectric micromanipulator (Burleigh Instruments, Victor, NY) and placed at approximately 100 μm in front of the central position of the stimulating electrode tips. After insertion of the carbon fibre electrode, the slice was allowed to recuperate for at least 5 minutes before starting recordings. Electrical stimulations were applied at intervals of 2 minutes. The first 10 minutes served as a control period. The peak of each response was measured and plotted over time.

### Drugs and chemicals

Chemicals were purchased from Sigma (St. Louis, MO) except for tetrodotoxin (TTX) (Alomone laboratories, Jerusalem, Israel). The 8–13 fragment of NT was used as a NT receptor agonist. It was used at a concentration of 100 nM, shown to produce maximal effects in previous studies [9]. The D2 receptor agonist quinpirole was used at a saturating concentration of 1 μM, while the antagonist sulpiride was used at a saturating dose of 5 μM, since numerous previous physiological studies use these ligands in the micromolar dose range [38-40]. Most drugs were stored in aliquots at -20°C as stock solutions and dissolved into aCSF immediately before use. Calcium, magnesium and glucose were freshly added to the aCSF, which was pre-oxygenated before use.

### Statistical analysis

Data are provided as mean ± SEM, with *n *representing the number of slices. Simple two group comparisons were performed using Student's *t*-test. Multiple group means were compared by analyses of variance (ANOVA), followed by a Tukey *post hoc *test. The minimal significance level for tests was set at p < 0.05. Graphs are expressed in percentage of control, where the control for each slice represents the average of [DA] recorded in normal saline before the entrance of the drug (T = 0 to T = 8). The response after a drug application was then analysed by comparing the maximal effect of the drug with the corresponding time of the control graph.

## List of abbreviations

NT: neurotensin; DA: dopamine; FCV: fast-scan cyclic voltammetry; VTA: ventral tegmental area; NAcc: nucleus accumbens; aCSF: artificial cerebrospinal fluid; TTX: tetrodotoxin; ANOVA: analysis of variance; NTR1: type 1 neurotensin receptor.

## Authors' contributions

CSF and PM and DL carried out the cyclic voltammetry experiments, performed data analysis and helped to draft the manuscript. LET conceived of the study and was responsible for the coordination of the project and helped to draft the manuscript. All authors read and approved the final manuscript.
